# An audit of vitamin D monitoring and management of deficiency in women's secure services

**DOI:** 10.1192/bjo.2021.91

**Published:** 2021-06-18

**Authors:** Claire Bustin, Shay-Anne Pantall, Jeremy Rampling

**Affiliations:** 1 University of Birmingham, Medical School; 2 Birmingham and Solihull Mental Health NHS Foundation Trust

## Abstract

**Aims:**

To audit the investigation, identification and treatment of vitamin D deficiency within Women's Secure Services.

**Background:**

It has been suggested that vitamin D and vitamin D deficiency may play a role in the pathogenesis of psychiatric illness. There is evidence that vitamin D inadequacy is prevalent among patients in long-term hospital settings. Patients within secure hospitals are considered to be at high risk due to their often lengthy admissions, having been transferred from other hospital or prison settings. Ardenleigh in Birmingham is a blended female secure unit. Here we present the findings of an audit, completed in 2019, of vitamin D monitoring and treatment in this service.

**Method:**

A retrospective review of electronic patient records, for all inpatients admitted within women's secure services at Ardenleigh as of 1st September 2019 (n = 27). Standards were based on the Trust accepted guidelines for management of vitamin D deficiency.

**Result:**

Key findings included:-

The majority of inpatients were Caucasian (44%) and African-Caribbean (41%). Median age was 31 years (range 20–56).

Approximately two-thirds (60%) had been in hospital for over a year.

89% of patients had their vitamin D level checked at some point during admission.

Of those checked, 25% were tested within 1 week of admission. Seven patients were tested after being in hospital for over one year (30%).

Only 25% of patients tested were found to have adequate vitamin D levels. Nine patients were found to have insufficient levels of vitamin D (37.5%) or deficiency (37.5%).

89% of those identified as requiring treatment were prescribed supplementation, of which the majority was prescribed at the correct dose for the appropriate duration (94%). One patient refused treatment. Of those with sufficient levels, 67% were prescribed ongoing maintenance treatment due to previously detected deficiency.

Of those found to have sufficient vitamin D in the last 12 months (n = 14), 71% were continued on maintenance treatment.

**Conclusion:**

We identified a high prevalence of vitamin D insufficiency in women admitted to secure services. Testing was delayed for a number of patients from the point of admission. However, once identified, the vast majority of those in need of treatment were managed appropriately by the medical team. We advise that vitamin D be considered an essential routine blood test at the point of admission to minimise delays in identifying those with deficiency and establishing necessary supplementation.

